# *In vivo* synergistic anti-tumor effect of paclitaxel nanoparticles combined with radiotherapy on human cervical carcinoma

**DOI:** 10.1080/10717544.2016.1230902

**Published:** 2017-02-03

**Authors:** YanXin Yu, Shan Xu, Hong You, YinJie Zhang, Bo Yang, XiaoYang Sun, LingLin Yang, Yue Chen, ShaoZhi Fu, JingBo Wu

**Affiliations:** 1 Department of Oncology and; 2 Department of Nuclear Medicine, the Affiliated Hospital of Southwest Medical University, Luzhou, China

**Keywords:** Paclitaxel, nanoparticle, radiotherapy, synergistic effect, cervical cancer

## Abstract

In this study, our purpose was to explore the synergistic anti-tumor effect and mechanism of paclitaxel nanoparticles (PTX-NPs) combined with radiotherapy (RT) on human cervical carcinoma (HeLa). PTX-NPs were prepared by a solid dispersion method using methoxy poly(ethylene glycol)–poly(ɛ-caprolactone) (MPEG–PCL), which combined with RT exerted a potent and high efficient effect against cervical cancer. *In vivo* antitumor activity of PTX-NPs combined with RT, was estimated using nude mice carrying Hela cell xenograft tumor. The results were evaluated using microfluorine-18-deoxyglucose PET/computed tomography (^18^F-FDG PET/CT) and immunohistochemistry. The results showed that PTX-NPs possessed a more efficient effect than PTX when combined with RT (*p* < 0.05). PTX-NPs in combination with RT might inhibit cell proliferation through its action on Ki-67, and decreased micro-vessel density (MVD) associated with CD31 and vascular endothelial growth factor (VEGF). These results suggested that PTX-NPs possessed a synergistic anti-tumor effect against cervical cancer when combined with RT.

## Introduction

Cervical cancer is the third most common malignant tumor in women worldwide and chemoradiotherapy is the most effective treatment in patients with locally advanced cervical cancer (Yee et al., 2013). Indeed, chemoradiotherapy improves local tumor control and the survival rate when compared to either continuous treatment or sole administration of chemotherapy or radiotherapy (RT) (Eifel, 2006; Seiwert et al., 2007). However, the synergistic use of both chemotherapy and RT has significantly higher systemic toxicity compared to either treatment alone or sequential use, due to the accumulative effect that the traditional chemotherapeutics fails to penetrate tumor interstitium in an effective concentration and increases the toxicity toward healthy tissues (Garrastazu Pereira et al., 2015; Wang et al., 2016), while the RT for cervical cancer can aggravate the side-effect causing the radiation damage to adjacent healthy tissues inevitably (Hafiz et al., 2015), such as hematological, urogenital and intestinal toxicity. Therefore, in order to reduce systemic toxicity and increase anti-tumor effect, a new drug delivery system of chemotherapy drug is in demand to the treatment of tumor. A new and exciting approach is to incorporate NPs into chemoradiotherapy. Drug delivery systems based on NPs have been extensively studied, and possess unique properties, making NPs the ideal device for the delivery of chemotherapy drugs in chemoradiotherapy, thus improving its therapeutic efficacy (Werner et al., 2011; Jung et al., 2012; Gong et al., 2013).

Paclitaxel (PTX) possesses antineoplastic activity against various types of solid tumors, verified radiosensitization, ability to inhibit microtubulin formation as well as endothelial cell proliferation and migration (Terasima & Tolmach, 1963; Downing, 2000; Pasquier et al., 2004,2005; Schwartz, 2009; Jiang et al., 2011; Werner et al., 2013; Zhang et al., 2014; Wang et al., 2016). However, PTX also possesses short circulation half-life, poor aqueous solubility, severe side effects due to its nonselective distribution *in vivo* and its intratumoral concentration is too low to cause mitotic arrest, all factors that often compromise its clinical efficacy (Yu et al., 2010; Weaver, 2014). In light of these obstacles, nanotechnology offers an alternative strategy to address the drug to tumor tissue using several nanoscaled drug delivery systems (Wang X et al., 2011; Huang et al., 2012; Lu et al., 2013a; Estanqueiro et al., 2015). The solubilization of PTX is more effective *in vitro* and *in vivo* than Taxol® in many solid tumors (Passarella et al., 2010; Xin et al., 2010; Guo et al., 2011; Yang et al., 2011; Jung et al., 2012; Lv et al., 2012; Yoshizawa et al., 2012; Zhang et al., 2012,2014; Lu et al., 2013b; Wu et al., 2014; You et al., 2016). In addition, with the development of nanotechnology, methoxy poly(ethylene glycol)–poly(ɛ-caprolactone) (MPEG–PCL) polymeric micelle appears a promising drug carrier, since it is biodegradable, effective, and possesses sustained drug release properties (Wang C et al., 2011; Ding et al., 2012; Gong et al., 2013; Wu et al., 2014; You et al., 2016). Its hydrophobic surface (PCL chains) forms a core for attaching hydrophobic drugs, while the hydrophilic one (PEG chains) forms a shell to act as a stabilizing interface (Gong et al., 2010). Such core–shell structure with the drug in the core effectively protected, ensures human body a low toxicity and a prolonged circulation in the blood due to its high water-solubility (Kataoka et al., 2001). In addition, the passive accumulation of the micelles in a solid tumor is achieved by the so-called enhanced permeation and retention (EPR) (Matsumura & Maeda, 1986; Xie et al., 2007). Therefore, hydrophobic drugs can be encapsulated into the MPEG–PCL micelles and can form a stable water-based formulation for potential clinical applications.

However, no reports are available on the therapeutic effects of MPEG–PCL/PTX polymeric micelles combined with RT against cervical cancer. Therefore, the purpose of this study was to improve the efficacy of chemoradiation therapy by developing Cremophor® EL-free NPs of MPEG–PCL loaded with PTX. The PTX nanoparticles (PTX-NPs) were characterized in terms of size and surface morphology. *In vitro* drug release was evaluated by dialysis method. Finally, *in vivo* tumor growth inhibition was analyzed in BABL/c tumor bearing mice.

## Materials and methods

### Materials, cells and animals

ɛ-Caprolactone (ɛ-CL) (Alfa Aesar, Ward Hill, MA), poly(ethylene glycol) methyl ether (MPEG, Mn = 2000, Aldrich, St. Louis, MO), stannous octoate (Sn(Oct)2) and 3-(4,5-dimethyl-2-thiazolyl)-2,5-diphenyl-2H-tetrazolium bromide (methyl thiazolyltetrazolium, MTT) were purchased from Sigma-Aldrich, St. Louis, MO. Paclitaxel with 99.8% purity was purchased from Chengdu Man Site Biotechnology Co. Ltd. (Chengdu, China). PTX 5 ml: 30 mg injectable solution (Taiji Group Co., Ltd., Chengdu, China) was supplied by Southwest Medical University. Dimethyl sulfoxide (DMSO), methyl alcohol and acetonitrile (HPLC grade) were supplied by Kelong Co. Ltd. (Chengdu, China). Ki-67, CD31 and VEGF polyclonal antibodies were purchased from Bioworld Technology Co. Ltd. (Nanjing, China).

Human cervical carcinoma cells (HeLa) were provided by the Experimental Medicine Center, at the Affiliated Hospital of Southwest Medical University (Luzhou, China). HeLa cells were grown in 25 cm^2^ glass culture flasks dissolved in 5 ml DMEM supplemented with 10% (v/v) fetal bovine serum (FBS) and 100 IU/ml of penicillin G sodium and 100 μg/ml of streptomycin sulfate, stored in an incubator at 37 °C with 5% CO_2_/95% air humidified atmosphere, subcultured three times per week and used for the experiments when in exponential growth phase.

Female athymic BALB/C nude mice weighing 14–18 g (3–4 weeks) were purchased from Chongqing Tengxin Biotechnology Co. Ltd. (Chongqing, China). Mice were housed at a controlled temperature of 20–22 °C, with a 50–60% relative humidity, fed with a standard laboratory chow and tap water *ad libitum*. All animals were handled in strict accordance with good animal practice as defined by the relevant national and/or local animal welfare bodies, and in accordance with the recommendations in the Guide for the Care and Use of Laboratory Animals of the National Institutes of Health. Animal experiments were approved by the Institutional Animal Care and Treatment Committee of Southwest Medical University (Luzhou, China).

Radiation was delivered at room temperature using a linear accelerator source (Medical Electronic Linear Accelerator, DRECISE-NET, Elekta, Atlanta, GA). The dose rate depended on the distance from the source. Radiation was delivered at 266.82 cGy/min for 1 min 52 s to produce a dose of 5 Gy.

### Preparation of PTX-NPs

Methoxy poly(ethylene glycol)–poly(ɛ-caprolactone) (MEP, *M*_w_ = 4 kDa) used in this study was synthesized by ring-opening polymerization of ɛ-CL on MPEG using Sn(Oct)_2_ as catalyst, according to a previous report (Wei et al., 2009). The PTX-NPs of free PTX at the dosage of 6%, 8% or 10% (w/w) were prepared by a solid dispersion method using MPEG–PCL according to a previous report (Wang C et al., 2011).

### Drug-loading (DL) and encapsulation efficiency (EE) of PTX-NPs

The drug loading and EE were determined in triplicate by a high performance liquid chromatography instrument (HPLC, Agilent 1260, Agilent Technologies, Santa Clara, CA), using a reverse phase C18 column (4.6 × 150 mm, 3.5 μm particle size). HPLC was calibrated using standard solutions of 5–100 μg/ml PTX dissolved in acetonitrile (correlation coefficient *R*^2^=0.9998). The quantification limit was 0.6 ng/ml. The coefficients of variation (CV) were all within 4.3%. The column temperature was maintained at 25 °C. Sample solution was injected at a volume of 50 μl. PTX-NPs were first dissolved in acetonitrile to destroy their package status, filtered through a 220 nm filter to obtain a sterile solution, and then the solution was measured at 227 nm. Methyl alcohol/acetonitrile/water (38.1/23.8/38.1, v/v/v) was used in its mobile phase with a flow rate of 1.0 ml/min. Drug-loading and EE were calculated using the following equations, as previously described (Wu et al., 2014):

$$\mathrm{DL}\%=\frac{\mathrm{Drug}}{\left(\mathrm{Polymer}+\mathrm{Drug}\right)}\times 100\%$$

$$\mathrm{EE}\%=\frac{\mathrm{Actual}\;\mathrm{drug}\;\mathrm{loading}}{\mathrm{Theoretical}\;\mathrm{drug}\;\mathrm{loading}}\times 100\%$$

### Physicochemical characterization and stability study of PTX-NPs

The average particle size and size polydispersity of prepared PTX-NPs in distilled water were measured by dynamic light scattering (DLS, NanoBrook 90Plus Zeta, Brookhaven, USA) at 25 °C. PTX-NPs (DL = 8%) were chosen according to results obtained and its detailed characterization was performed. The surface morphology of the PTX-NPs was investigated by transmission electron microscope (TEM, Tecnai G2 F20, Hillsboro, OR). PTX-NPs were diluted in distilled water and placed on a copper grid covered with nitrocellulose. PTX-NPs were negatively stained with phosphotungstic acid and dried at room temperature. Moreover, the formulation stability of the PTX-NPs at a concentration of 0.2 mg/ml in PBS and 10% FBS-containing PBS, kept at 4 °C or 25 °C, was evaluated by DLS after ultrasonic processing. Size changes were measured at pre-determined time points and the measurement was terminated when the change of size reached a significant difference. Formulations were judged stable if no changes in particle size and no visual destabilization were observed, such as creaming, phase separation, or presence of compact aggregates. All results represent the mean of three test runs and all data were expressed as mean ± standard deviation (SD).

### *In vitro* drug release

To measure PTX release profile from PTX-NPs, 1.0 ml PTX-NPs solution (theoretical drug loading rate was 8%) and 1.0 ml free PTX solution in dehydrated alcohol at a concentration of 0.5 mg/ml were placed in dialysis bags (molecular mass cutoff was 8.0–14 kDa). The dialysis bags were incubated in 40 ml PBS (pH = 7.4) containing Tween 80 (0.5%, w/v) at 37 °C with gentle shaking (100 rpm). At pre-determined time points, 2 ml aliquots of the dialysis medium were collected, and the same volume of fresh pre-warmed medium was added. The collected medium was centrifuged at 12 000 rpm for 15 min at 4 °C. The supernatant was collected and stored at −20 °C for further analysis. All samples were measured using HPLC as described in section Drug-loading (DL) and encapsulation efficiency (EE) of PTX-NPs, and data represent the mean of a triplicate and expressed as mean ± SD.

### *In vivo* anti-tumor efficacy of RT combined with PTX-NPs

Athymic BALB/C nude mice were subcutaneously treated in the right flank with 0.1 ml of Hela cell suspension containing 1 × 10^6^ cells. Administration of the compounds listed below started at day 15 after inoculation when the tumor size reached approximately 200 mm^3^. On that day, mice were randomly divided into six groups: Normal saline (NS) representing the control group, PTX, PTX-NPs, RT, PTX + RT and PTX-NPs + RT group (*n* = 12). NS infusion 0.9%, PTX injection or PTX-NPs solution (0.5 mg/ml PTX 2.5 mg/kg) were intravenously injected and subsequently tumor irradiation was performed six hours post injection. The dose rate at a source–subject distance of 70 cm was 60 cGy/min. Mice were irradiated to a total dose of 15 Gy with five daily fractions of 3 Gy. Mice head and abdomen regions were protected by 0.5 cm thick lead shielding. Only the truncal region containing tumor xenograft was irradiated. Fifty percent of the animals of each group were randomly euthanized 24 h after treatment; the remaining mice in each group were used to investigate the tumor regrowth delay (TGD) and animal survival. Animals’ tumor volumes and body weight were measured every three days using the formula as previously described (You et al., 2016): *V* = 0.5×*a*×*b*^2^, where *V* is the tumor volume, *a* is the length of the major axis and *b* is the length of the minor axis.

### Micro ^18^F-FDG PET/CT imaging

To investigate the tumor tissues’ metabolism early response in Control, PTX + RT and PTX-NPs + RT group, we performed micro PET/CT scans and image analysis using an Inveon micro PET/CT (Siemens, Munich, Germany) animal scanner at one day after the end of treatment. Mice were fasted 12 h, intraperitoneal anesthetized with 1% pentobarbital 5 ml/kg and 100–200 μCi FDG was intravenously injected in the tail prior to scan. Mice were positioned in the center PET ring field of view and PET/CT images were acquired 1 h after ^18^F-FDG administration. The parameters used were as follows: 80 kV, 500 μA, 1.5 mm slice thickness and 10 min per bed position.

According to a previous report (You et al., 2016), the image plane with the largest tumor appearance on the PET/CT fusion image was selected for data collection. An irregular region of interest (ROI) covering the entire tumor was manually drawn. ROIs were also drawn on the contralateral paraspinal muscles. Tracer uptake value in tumor and muscle tissue was determined in the attenuation-corrected transaxial tomographic slices by calculating the standard uptake value (SUV) and was measured by means of ROI. ^18^F-FDG maximum SUV of each lesion was obtained from the selected ROI and compared to the SUVs of the contralateral paraspinal to calculate the tumor/muscle (T/M) ratio.

### Ki-67, CD31 and VEGF immunohistochemistry

Tumor tissues were harvested and fixed with 4% formaldehyde. Next paraffin-embedded 4-μm thick sections were cut for hematoxylin and eosin (H&E) histological staining and immunohistochemistry. Briefly, tissues were incubated with anti-human Ki-67, CD31 and VEGF, primary antibodies, and a biotinylated goat anti-mouse antibody was used as secondary antibody. Then the slides were washed with PBS and incubated with diaminobenzidine (DAB) chromogen for 3–5 min to yield a dark brown color. The sections were counter-stained with hematoxylin for microscopic observation (Leica TE2000-S Microscope, Tokyo, Japan).

Ki-67 and VEGF expressions were calculated in five randomly selected areas in each tumor sample as the number of positive cells/total were counted at 400× magnification. Cells with moderate and strong brownish cytoplasmic staining were considered as positive, whereas cells with unstained or weakly stained cytoplasm were considered as negative.

Micro-vessel density (MVD) was determined as previously reported (Makitie et al., 1999) and expressed as the mean value of the area of microvessels in five CD31-highly positive hotspots.

### Statistical analysis

The statistical analysis was carried out using SPSS 13.0 software (Chicago, IL) and performed by Student’s *t*-test for two groups or one-way ANOVA for multiple groups. Means were considered different when *p < *0.05.

## Results

### PTX content and PTX-NPs physicochemical characterization

**Figure 1.**
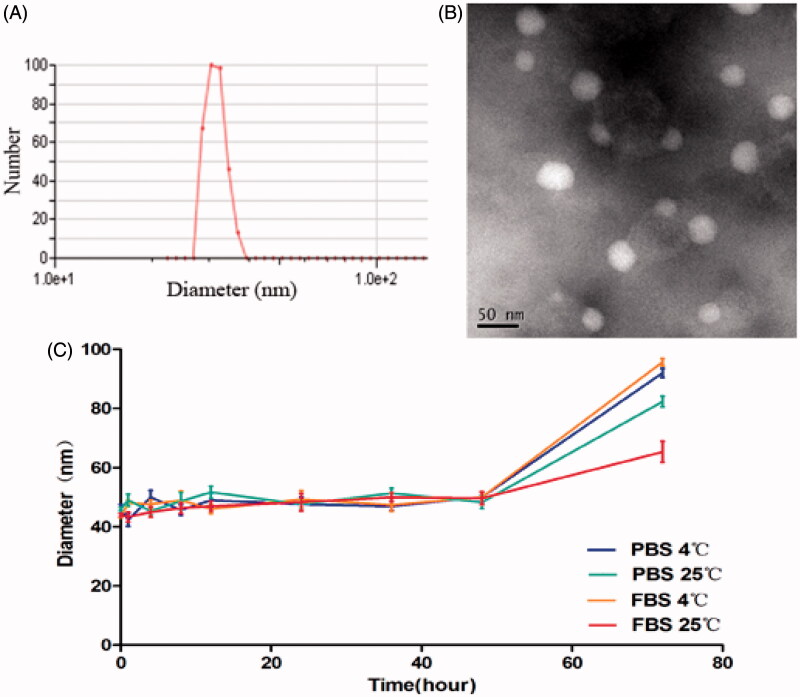
Physicochemical characterization and stability study of PTX-NPs. (A) PTX-NPs particle size distribution; (B) TEM image of PTX-NPs and (C) the formulation stability of the PTX-NPs. Data are shown as means ± SD (*n* = 3).

In this study, the theoretical DL into PTX-NPs was set at 6%, 8% and 10% (wt%). According to DL%, EE% and particle size (Table 1), 8% (7.96 ± 0.06) showed a better actual drug loading than 6% (4.33 ± 0.12) and 10% (8.79 ± 0.17). Therefore, we considered PTX-NPs 8% theoretical DL in our further experiments. The size and dispersity of prepared PTX-NPs were confirmed by TEM morphology (Figure 1A,B), showing well-dispersed nanoparticles into an aqueous solution. The formulation stability of PTX-NPs as shown in Figure 1(C), stayed stable for 48 h in each group without using any stabilizer.

**Table 1.** PTX content in nanoparticles.

Samples	Theoretical DL (%)	DL (%)	EE (%)	Size (nm)	PDI
1	6%	4.33 ± 0.12	92.76 ± 0.29	43.34 ± 0.27	0.21 ± 0.04
2	8%	7.96 ± 0.06	99.78 ± 0.34	45.71 ± 0.32	0.19 ± 0.02
3	10%	9.79 ± 0.17	97.90 ± 0.17	49.36 ± 0.40	0.20 ± 0.05

### *In vitro* drug release

**Figure 2.**
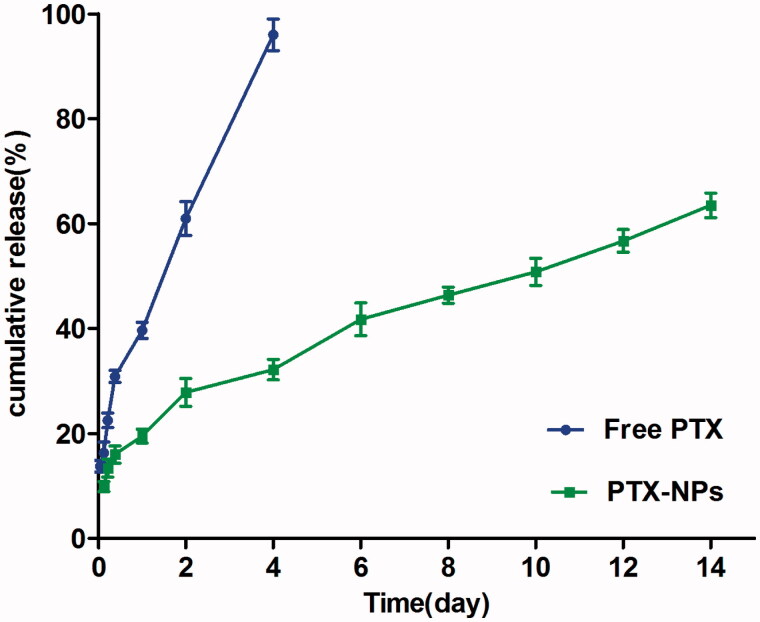
*In vitro* drug release of PTX-NPs and free PTX. Data are shown as means ± SD (*n* = 3).

Free PTX exhibited a rapid release behavior, and more than 97% of the drug was released within four days. However, only 63.48% PTX was slowly released from the PTX-NPs in 14 days, with no burst effect, as compared to free PTX (Figure 2).

### *In vivo* tumor growth inhibition

**Figure 3.**
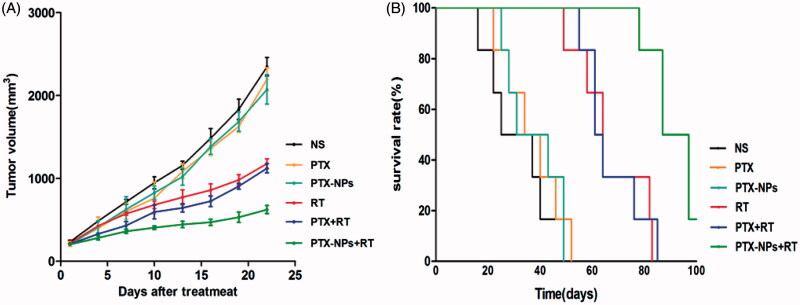
PTX-NPs + RT inhibited tumor growth in subcutaneous HeLa model. (A) Suppression of subcutaneous tumor growth in each group. (B) Mice survival curve in each group.

To evaluate the efficacy of PTX-NPs combined with RT on HeLa xenograft in mice, tumor volume and median survival time were measured and plotted after each treatment. After 22 days, the tumor volume of PTX-NPs + RT group (623.5 mm^3^) was significantly smaller than PTX + RT group (1121.4 mm^3^, *p* < 0.01), RT group (1176.9 mm^3^, *p* < 0.01), PTX-NPs group (2074.1 mm^3^, *p* < 0.01), PTX group (2197.2 mm^3^, *p* < 0.01) and NS group (2347.2 mm^3^, *p* < 0.01) (Figure 3A). These results demonstrated that PTX-NPs + RT possessed a more powerful ability on reducing the cervical tumor volume.

Mice median survival time (Figure 3B) in the PTX-NPs + RT group was significantly longer (87 days) compared to the PTX + RT group (61 days, *p* < 0.01), RT group (64 days, *p* < 0.01), PTX-NPs group (31 days, *p* < 0.01), PTX group (34 days, *p* < 0.01) and NS group (25 days, *p* < 0.01), suggesting that PTX-NPs was more effective, compared to PTX, when combined with RT. Body weight measurements showed no significant differences between the groups throughout the entire study. A slight increase in body mass was observed as a result of natural animal growth (data not shown).

### Micro ^18^F-FDG PET imaging

**Figure 4.**
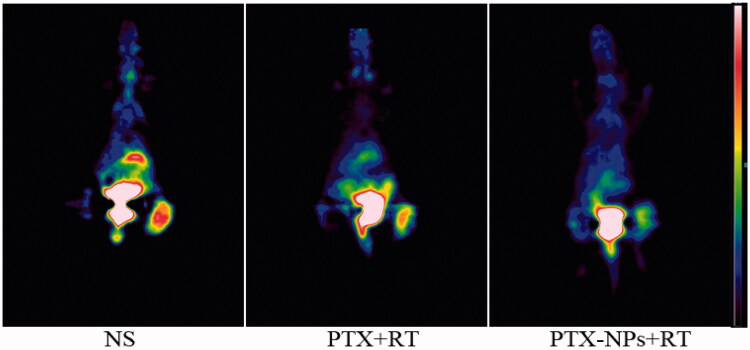
Representative 18F-FDG PET images of mice after one full day treatment.

The T/M values of each group were 2.635 ± 0.074 (NS), 2.032 ± 0.214 (PTX + RT) and 1.182 ± 0.043 (PTX-NPs + RT). Both PTX + RT and PTX-NPs + RT groups showed a decrease in T/M value compared to the NS group, although the T/M value in PTX-NPs + RT group was the lowest (*p *< 0.05), suggesting a more powerful effect of PTX-NPs, when combined with RT, than PTX + RT (Figure 4).

### Immunohistochemistry

**Figure 5.**
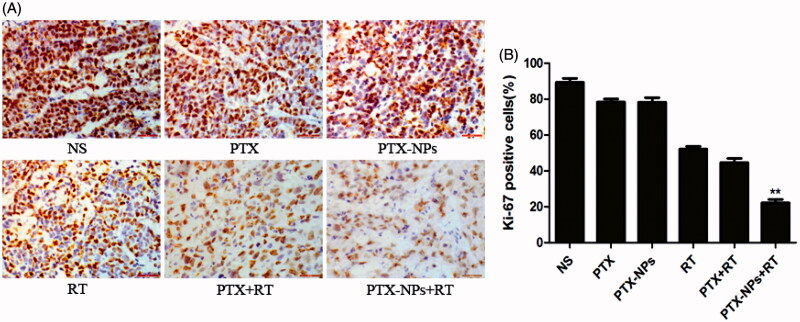
Ki-67 immunohistochemical staining in tumors. (A) Ki-67 immunohistochemical images of tumor tissue from mice in various groups. (B) Ki-67 quantitative analysis in xenografts from mice in various groups. *p < 0.05 and **p < 0.01. Original magnification, ×400.

A small number of Ki-67 positive cells was observed in the PTX-NPs + RT group (21.7 ± 7.5%) compared to PTX + RT group (40.6 ± 4.4%, *p* < 0.05), RT group (57.2 ± 5.0%, *p* < 0.05), PTX-NPs group (73.2 ± 5.8%, *p* < 0.05), PTX group (79.6 ± 6.2%, *p* < 0.05) or NS group (87.2 ± 4.0%, *p* < 0.05) (Figure 5).

**Figure 6.**
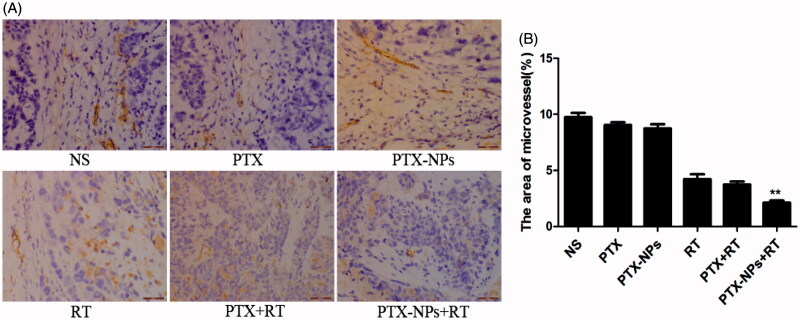
CD31 immunohistochemical staining of tumors. (A) CD31 immunohistochemical images of tumor tissue from mice in various groups. (B) CD31 quantitative analysis in xenografts from mice in various groups. *p < 0.05 and **p < 0.01. Original magnification, ×400.

Fewer immunoreactive microvessels were observed in the tumor tissue sections of PTX-NPs + RT treated mice (Figure 6). The area of microvessel percentage of the MVD area in the PTX-NPs + RT group (2.1 ± 0.3%) was dramatically lower compared to the PTX + RT group (3.8 ± 0.4%, *p* < 0.05), RT group (4.2 ± 0.8%, *p* < 0.05), PTX-NPs group (8.7 ± 0.7%, *p* < 0.05), PTX group (9.0 ± 0.4%, *p* < 0.05) or NS group (9.8 ± 0.7%, *p* < 0.05).

**Figure 7.**
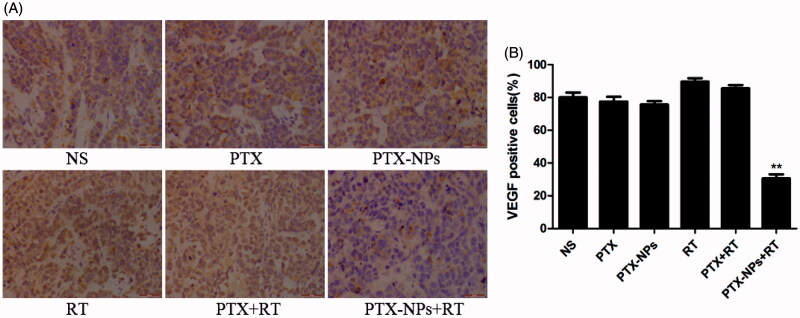
VEGF immunohistochemical staining in tumors. (A) VEGF immunohistochemical images of tumor tissue from mice in various groups. (B) VEGF quantitative analysis in xenografts from mice in various groups. *p < 0.05 and **p < 0.01. Original magnification, ×400.

The expression of VEGF protein in the PTX-NPs + RT group was 30.7 ± 4.0%, that was actually significantly lower than that of the PTX + RT group (85.6 ± 3.5%, *p* < 0.01), RT group (89.7 ± 3.5%, *p* < 0.01), PTX-NPs group (75.7 ± 3.2%, *p* < 0.01), PTX group (77.4 ± 5.1%, *p* < 0.01) or NS group (80.0 ± 5.0%, *p* < 0.01, Figure 7).

## Discussion

In the present work, the method we used to obtain PTX-NPs allowed us to obtain nanoparticles with a specific stability, EE and drug loading according to previous studies (Wang C et al., 2011). The most important aspect of these results was that the obtained size prevents nanoparticles renal filtration during circulation in the human body, thus increasing the blood circulation time and facilitating an effective and powerful targeting of the tumor through the EPR effect (Xu et al., 2015). In addition, although it was kept stable for a much shorter time (48 h) without any stabilizing agent compared to the study using soybean LEC for stabilization (Kumar et al., 2014), the lyophilized nanoparticles could be homogeneously and stably dissolved, as previously reported (Wu et al., 2014), ensuring the best solubility into the blood and on the same time, and they were more suitable for storage and large-scale production.

The sustained and slow drug release exhibited by PTX-NPs could be due to the stable structure of polymeric micelles, since it might be attributed to the diffusion of the drug localized in the PCL core of the nanoparticles, suggesting that our PTX-NPs had an excellent drug loading and EE. The rapid drug release by free PTX indicated that the dialysis membrane had no effect on retarding the release of the drug from nanoparticles, also underlining that PTX-NPs drug release mechanisms are diffusion, erosion and swelling of the polymer matrix, as well as its degradation (Zhang & Feng, 2006). The burst release of PTX in other studies (Wu et al., 2014) may be due to the dissolution and diffusion of the drug that was poorly entrapped in the polymer matrix. Some studies have reported that the NPs cumulative drug release within one month is usually 30–40% or less, which may be too slow to meet the therapeutic needs (Mu & Feng, 2002; Zhang & Feng, 2006). Thus, our PTX-NPs possessed the great advantage of an increased drug release rate. This can be due to the PEG content in the material, giving to the NPs a hydrophilic character, leading to a faster release of the drug. Such drug release we obtained could thus allow an increased synergistic effect between PTX and RT, which in turn could result in an improved therapeutic efficacy.

*In vivo* experiments, we demonstrated that at equivalent PTX doses, PTX-NPs led to a significantly longer delay on tumor growth and the survival time than PTX injection in mice treated with chemoradiation therapy. It is important to note that in this study we used a relatively low dose of PTX, suggesting that PTX-NPs paclitaxel was more effective than PTX on cervical cancer due to the EPR of NPs.

According to a previous study (Avril et al., 2011), a higher FDG uptake in tumors suggested a poor response and prognosis while a lower FDG uptake may indicate a better response to treatment. Our result showing the lowest ^18^F-FDG uptake by PTX-NPs + RT group suggested that PTX-NPs enhanced RT effect to a greater extent compared to PTX injection.

Furthermore, the percentage of Ki-67 positive cells in each groups exhibited an analogous difference in the tumor growth delay and mice median survival time. In addition, a similar difference was also found in the MVD area and CD31-positive vessels, suggesting that PTX-NPs combined with RT might contribute to the suppression of cell proliferation and angiogenesis

However, compared PTX + RT group to RT group, although the expression of VEGF had no statistically significant as the expression of Ki-67 or CD-31, the expression of VEGF was higher than other non-RT groups. VEGF is a protein secreted by tumor cells to promote angiogenesis through proliferation, migration, and the survival of endothelial cells acting as an antiapoptotic factor. It also involved in cervical cancer (Stepan et al., 2012; Wachsberger et al., 2012; Barbu et al., 2013; Du et al., 2014; Shi et al., 2015). Thus, VEGF elevated expression as we observed in RT alone or with PTX might contribute to the resistance to radiation in tumors (Zhou et al., 2012). On the other hand, VEGF in the PTX-NPs + RT group reached the lowest expression, compared to the other groups, suggesting that PTX-NPs play a role in decreasing VEGF against the increased level of RT, which in turn suggest that PTX released from NPs modulated the interaction between the tumor and its microenvironment due to the EPR of NPs. Furthermore, the VEGF, which is associated with MVD, influenced by RT might be instantaneous, and the decreased area of microvessel contributed to the directly destroy of RT and the EPR of PTX-NPs.

## Conclusions

In this study, a NPs drug delivery system composed of PTX micelles was prepared and its *in vivo* anti-tumor activity was investigated in a mouse model for cervical cancer. The PTX-NPs were prepared by solid dispersion method and produced a compound with high DL, high EE, spherical morphology and monodispersity. *In vitro* drug release experiments showed that the PTX-NPs could release PTX in a controlled manner. The *in vivo* assay showed that the PTX-NPs at a low dose combined with RT can significantly inhibit tumor growth and prolong the survival time of tumor-bearing mice. PET/CT imaging on the HeLa xenograft model also verified that PTX-NPs enhanced the effect of RT compared to PTX injection. Analysis of tissue biomarkers showed that the PTX-NPs combined with RT suppressed tumor cell proliferation and inhibited angiogenesis in tumor tissue. Thus, the results indicated that PTX-NPs had an obviously efficient for cervical cancer without an accumulation antitumor efficient of PTX but a synergism of PTX combined with RT.
